# Gender-specific associations of vision and hearing impairments with adverse health outcomes in older Japanese: a population-based cohort study

**DOI:** 10.1186/1471-2318-9-50

**Published:** 2009-11-22

**Authors:** Takehiro Michikawa, Yuji Nishiwaki, Yuriko Kikuchi, Makiko Nakano, Satoko Iwasawa, Keiko Asakura, Ai Milojevic, Kunio Mizutari, Hideyuki Saito, Susumu Ishida, Tomonori Okamura, Toru Takebayashi

**Affiliations:** 1 Department of Preventive Medicine and Public Health, School of Medicine, Keio University, 35 Shinanomachi, Shinjuku-ku, Tokyo 160-8582, Japan; 2 Department of Otorhinolaryngology, Head and Neck Surgery, School of Medicine, Keio University, 35 Shinanomachi, Shinjuku-ku, Tokyo 160-8582, Japan; 3 Department of Ophthalmology, Hokkaido University Graduate School of Medicine, Kita 14, Nishi 5, Kita-ku, Sapporo 060-8648, Japan; 4 Department of Preventive Cardiology, National Cardiovascular Center, 5-7-1 Fujishirodai, Suita, Osaka 565-8565, Japan

## Abstract

**Background:**

Several epidemiological studies have shown that self-reported vision and hearing impairments are associated with adverse health outcomes (AHOs) in older populations; however, few studies have used objective sensory measurements or investigated the role of gender in this association. Therefore, we examined the association of vision and hearing impairments (as measured by objective methods) with AHOs (dependence in activities of daily living or death), and whether this association differed by gender.

**Methods:**

From 2005 to 2006, a total of 801 residents (337 men and 464 women) aged 65 years or older of Kurabuchi Town, Gunma, Japan, participated in a baseline examination that included vision and hearing assessments; they were followed up through September 2008. Vision impairment was defined as a corrected visual acuity of worse than 0.5 (logMAR = 0.3) in the better eye, and hearing impairment was defined as a failure to hear a 30 dB hearing level signal at 1 kHz in the better ear. Information on outcomes was obtained from the town hall and through face-to-face home visit interviews. We calculated the risk ratios (RRs) of AHOs for vision and hearing impairments according to gender.

**Results:**

During a mean follow-up period of 3 years, 34 men (10.1%) and 52 women (11.3%) had AHOs. In both genders, vision impairment was related to an elevated risk of AHOs (multi-adjusted RR for men and women together = 1.60, 95% CI = 1.05-2.44), with no statistically significant interaction between the genders. In contrast, a significant association between hearing impairment and AHOs (multi-adjusted RR = 3.10, 95% CI = 1.43-6.72) was found only in the men.

**Conclusion:**

In this older Japanese population, sensory impairments were clearly associated with AHOs, and the association appeared to vary according to gender. Gender-specific associations between sensory impairments and AHOs warrant further investigation.

## Background

It is not well known that adult-onset hearing loss and refractive errors (age-related causes of vision impairment) are respectively the world's highest- and second highest-ranking disabilities among people aged 60 years and over [1]. This lack of awareness means that sensory impairments have been largely dismissed as non-fatal, inevitable aspects of aging and have received minimal attention. There is growing evidence that self-reported vision and hearing impairments are associated with adverse health outcomes (AHOs) in older populations, and sensory impairments are likely to become an important public health issue [2-7]. To date, few studies have used objective vision and hearing examinations to investigate the association between sensory impairments and AHOs [8,9].

Evidence of gender differences has been reported regarding many aspects of health in older populations [10]. Women have a longer life expectancy than men in most countries, including Japan [11]. In contrast, women have a higher prevalence of co-morbid conditions than men [12,13]. All the evidence suggests the need for further research that pays attention to gender differences instead of treating gender as a confounder. To begin with, the prevalence of sensory impairments differs between men and women. Earlier studies showed that hearing impairment is more prevalent among men than among women, while women had a higher prevalence of vision impairment [14-16]. In addition, Appollonio et al. reported that in a cohort study in Italy the association of sensory impairments with mortality was gender-specific [17]. Thus, it is reasonable to assume that the association between sensory impairments and AHOs also differs according to gender. Even so, little research has been done on the question of gender differences.

With these points in mind, we tried to clarify the association of vision and hearing impairments (as measured by objective methods) with AHOs (dependence in activities of daily living [ADLs] or death), by analyzing 3-year follow-up data from a population-based cohort study of community-dwelling older Japanese. Our hypothesis was that the association would vary by gender.

## Methods

### Study population

The Kurabuchi study is an ongoing community-based longitudinal study of aging involving functional assessment of an older population in Kurabuchi Town, Takasaki City (approximately 100 km north of Tokyo, Japan) [18-20]. Between 2005 and 2006, public health nurses and local welfare commissioners visited home of residents aged 65 years or older and collected health information in face-to-face interviews. We identified 1,294 non-institutionalized, functionally independent residents as eligible subjects. All of them were invited to participate in detailed health assessments at 8 community centers. Of the 1,294 eligible residents, 801 (337 men and 464 women; 62%) took part in the detailed health assessments, and were followed for about 3 years until September 2008.

Comparisons between the participants and non-participants have been published previously [20]. In brief, the participants included a higher proportion of women and tended to be younger and better educated than the non-participants. On the other hand, there were no significant differences between the 2 groups in terms of difficulty reading a newspaper or hearing.

The Ethics Committee of the School of Medicine, Keio University (Tokyo, Japan) approved this study, and written informed consent was obtained from all participants.

### Sensory examinations at baseline

Corrected distance (5 m) visual acuity, widely used in research into the association between vision impairment and AHOs [8,9,17,21], was measured by trained technicians with a Landolt broken ring chart using an automatic visual analyzer (TOMEY NS-1100, Nagoya, Japan) under standard lighting conditions. We defined vision impairment as a corrected visual acuity of worse than 0.5 (logMAR = 0.3) in the better eye, following the US criteria for visual impairment [8,9]. History of cataract surgery was also ascertained.

Pure-tone air-conduction audiometry was conducted in a separate quiet room by trained technicians with an audiometer (AA-56, RION Inc., Tokyo, Japan); subjects who used hearing aids were asked to remove them for the tests. To reduce the influence of ambient noise (24 to 29 dB sound pressure level [A]), circumaural earphones were used. The audiometer was calibrated regularly to comply with Japanese standards. Due to the field setting nature of the study, we tested a signal with an intensity of 30 dB hearing level (HL) at a frequency of 1 kHz, and a signal with an intensity of 40 dB HL at a frequency of 4 kHz, in accordance with Japan's Industrial Safety and Health Law's stipulations on health examinations for workers. Since daily conversation in Japanese relies more on low-frequency signals than high-frequency signals [22], we defined hearing impairment as a failure to hear a 30 dB HL signal at 1 kHz in the better ear [20]. We also obtained information on hearing aids usage.

### Covariates' information at baseline

Body mass index (BMI) was calculated as weight (kg) divided by the square of height (m). Trained staffs measured blood pressure with an automatic sphygmomanometer in the right arm of seated participants after at least 15 minutes of rest. We defined hypertension as systolic blood pressure ≥ 140 mmHg and/or diastolic blood pressure ≥ 90 mmHg and/or use of antihypertensive agents. Non-fasting blood samples were taken for measurements of total cholesterol (enzymatic method), albumin (nephelometry method), hemoglobin A_1c _(high-performance liquid chromatographic method), and C-reactive protein (latex turbidimetric immunoassay). Subjects were classified as having diabetes mellitus if it was self-reported and/or they had a hemoglobin A_1c _level ≥ 5.8%. Information was also collected on the following: age; educational level (elementary or junior high school, high school or higher); marital status (married, single/divorced/widowed); smoking status (current smoker or not); alcohol consumption (current drinker or not); occupational noise exposure (yes, no); past/current history of major illness including stroke, coronary heart disease, cancer or Parkinson disease (summary of yes or no); and knee joint pain (yes, no). Additionally, we used well validated scales to evaluate the following 3 items as potential intermediate factors linking sensory impairments and AHOs: instrumental activities of daily living (IADLs) (Tokyo Metropolitan Institute of Gerontology Index of Competence [TMIG-IC], score 0-13)) [23]; depressive mode (Geriatric Depression Scale, 5-item version [GDS5], score 0-5) [24], and cognitive function (clock drawing test [CDT], score 0-4)) [25].

### Outcome measurements

We defined AHOs [26] as either dependence in ADLs or death, i.e. the final form of functional transition [27]. Dependence in ADLs included nursing home admission or "long-term care" (LTC) eligibility, or impaired basic ADLs. LTC insurance was implemented by the Japanese government in 2000 to provide support according to the specific care needs of older people [28]. Those who want such support apply to their local town hall, where an initial assessment is made by computer of an 82-item questionnaire on physical and mental status. A board of medical and social services experts then uses this initial assessment, together with information provided by the assessor and attending doctor, to determine eligibility.

Information about death, nursing home admission, and LTC eligibility was obtained from the Kurabuchi Branch Office of Takasaki City Hall. To collect information about basic ADLs according to the Katz Index [29], repeat face-to-face home visit interviews were conducted twice (in 2007 and 2008). Participants were asked about their ability to perform the 6 basic ADL items (feeding, dressing, bathing, going to toilet, continence, and transferring [moving in and out of bed as well as in and out of chair]), and were given 3 response options: without help, with partial help, or with full help. We defined impaired basic ADLs as partial or full help needed to perform any of the 6 items.

### Statistical analysis

All analyses were performed with STATA version 9 (STATA Corporation, College Station, Texas) after stratification by gender to test our hypothesis.

The chi-square test was used to compare the prevalence of baseline characteristics in relation to vision and hearing impairments. We used a Poisson regression with a robust error variance [30] to estimate risk ratios (RRs) and 95% confidence intervals (CIs) as a measure of the relationship between vision/hearing impairments and AHOs. After calculation of crude RRs, a model mutually adjusted for vision and hearing impairments (Model 1) was applied. We then adjusted for age, educational level, marital status, smoking, alcohol consumption, past/current history of major illness and diabetes mellitus in addition to Model 1 (Model 2), since each of these variables changed the effect estimates by more than 10% [31] in an age-adjusted model. By adding the omitted variables, including history of cataract surgery, to the final model one by one, we confirmed that they did not alter the effect estimates. We examined the statistical interaction between sensory impairments and gender with a likelihood ratio test. To evaluate reverse causation between sensory impairments and AHOs, we also calculated the RRs after excluding events occurring within the first 1 year of the study. We found no interaction between vision/hearing impairments on AHOs in this study.

IADLs, depressive mode and cognitive function were associated with AHOs in our study population. These three variables could be considered as intermediate factors linking sensory impairments and AHOs [6,9,20,21,32]. Therefore, RRs were compared between the genders with 3 additional models including respectively IADLs (TMIG-IC score), depressive mode (GDS5 score), and cognitive function (CDT score).

## Results

Of the 801 participants, we followed 796 (5 residents moved out of Kurabuchi) (follow-up proportion = 99.4%). During the 3-year follow-up period, 34 men (10.1%) and 52 women (11.3%) had AHOs (52 became dependent in ADLs and 34 died).

The baseline characteristics of the participants are grouped by gender and according to vision and hearing impairments in Table 1. Age distribution, educational level, IADLs, depressive mode and cognitive function tended to differ between the impaired and unimpaired groups, regardless of gender. There were no significant differences in the proportion of current smoker, elevated BMI, high total cholesterol level, low albumin level, or past/current history of major illness and diabetes between the 2 groups.

**Table 1:** Baseline characteristics (2005-2006) of the subjects according to gender and sensory impairments.

	Men				Women			
	**Vision impairment**		**Hearing impairment**		**Vision impairment**		**Hearing impairment**	
	**No**	**Impaired**	**No**	**Impaired**	**No**	**Impaired**	**No**	**Impaired**
**Variables**	**(n = 275)**	**(n = 60)**	**(n = 271)**	**(n = 64)**	**(n = 301)**	**(n = 160)**	**(n = 345)**	**(n = 116)**
	**Number (%)**	**Number (%)**	**Number (%)**	**Number (%)**	**Number (%)**	**Number (%)**	**Number (%)**	**Number (%)**

Age (65-69 years)	68 (24.7)	6 (10.0)	71 (26.2)	3 (4.7)	80 (26.6)	20 (12.5)	91 (26.4)	9 (7.8)
(70-79 years)	152 (55.3)	32 (53.3)	152 (56.1)	32 (50.0)	163 (54.2)	80 (50.0)	190 (55.1)	53 (45.7)
(80- years)	55 (20.0)	22 (36.7)*	48 (17.7)	29 (45.3)*	58 (19.2)	60 (37.5)*	64 (18.5)	54 (46.5)*
Education (high school or higher)	78 (29.7)	8 (14.6)*	71 (27.8)	15 (24.2)	75 (25.5)	20 (12.7)*	76 (22.7)	19 (16.4)
Marital status (married)	221 (84.7)	46 (83.6)	216 (85.0)	51 (82.3)	179 (61.5)	83 (52.9)	204 (61.3)	58 (50.4)*
Current smoker (yes)	69 (26.1)	17 (30.4)	69 (26.9)	17 (27.0)	10 (3.4)	5 (3.1)	11 (3.3)	4 (3.5)
Current alcohol drinker (yes)	149 (56.9)	34 (60.7)	149 (58.4)	34 (54.0)	46 (15.8)	14 (8.9)*	48 (14.3)	12 (10.5)
Occupational noise exposure (yes)	97 (35.3)	23 (38.3)	90 (33.2)	30 (46.9)*	52 (17.3)	21 (13.1)	54 (15.7)	19 (16.4)
Body mass index (< 18.5 kg/m^2^)	15 (5.5)	5 (8.3)	18 (6.6)	2 (3.1)	28 (9.3)	14 (8.8)	36 (10.4)	6 (5.2)
(≥ 25.0 kg/m^2^)	77 (28.0)	8 (13.3)	72 (26.6)	13 (20.3)	83 (27.6)	35 (21.9)	84 (24.4)	34 (29.3)
Total cholesterol (≥ 5.7 mmol/L)	45 (16.4)	12 (20.3)	48 (17.7)	9 (14.3)	125 (41.5)	59 (36.9)	141 (40.9)	43 (37.1)
Albumin (≤ 3.8 g/dl)	22 (8.0)	2 (3.4)	20 (7.4)	4 (6.3)	12 (4.0)	7 (4.4)	11 (3.2)	8 (6.9)
C-reactive protein (> 0.3 mg/dl)	33 (12.0)	8 (13.6)	33 (12.2)	8 (12.7)	23 (7.6)	13 (8.1)	21 (6.1)	15 (12.9)*
Past/current history of major illness (yes) ^a^	57 (22.1)	14 (25.9)	54 (21.4)	17 (28.3)	40 (13.8)	30 (19.1)	46 (13.8)	24 (21.2)
Past/current history of hypertension (yes) ^b^	169 (63.5)	33 (60.0)	168 (64.6)	34 (55.7)	197 (66.6)	116 (73.0)	225 (66.2)	88 (76.5)*
Past/current history of diabetes mellitus (yes) ^c^	58 (22.3)	14 (24.6)	59 (23.1)	13 (21.3)	46 (15.9)	32 (20.3)	53 (15.8)	25 (22.1)
Past/current history of knee joint pain (yes)	85 (31.0)	28 (46.7)*	81 (29.9)	32 (50.8)*	129 (42.9)	71 (44.4)	145 (42.0)	55 (47.4)
History of cataract surgery (yes)	41 (14.9)	8 (13.3)	37 (13.7)	12 (18.8)	46 (15.3)	23 (14.4)	41 (11.9)	28 (24.1)*
Hearing aid usage (yes)	15 (5.5)	7 (11.7)	3 (1.1)	19 (29.7)*	8 (2.7)	6 (3.8)	2 (0.6)	12 (10.3)*
Instrumental activities of daily living (declined) ^d^	22 (8.4)	14 (25.5)*	27 (10.6)	9 (14.5)	21 (7.3)	39 (24.7)*	37 (11.2)	23 (19.8)*
Depressive mode (depressed) ^e^	61 (22.5)	17 (28.3)	54 (20.2)	24 (38.1)*	75 (25.3)	75 (46.9)*	101 (29.6)	49 (42.6)*
Cognitive function (impaired) ^f^	9 (3.3)	12 (20.0)*	14 (5.2)	7 (10.9)	5 (1.7)	16 (10.1)*	12 (3.5)	9 (7.8)

* P < .05 by the chi-square test. ^a^Past/current history of major illness contained stroke, coronary heart disease, cancer and Parkinson disease. ^b^Past/current history of hypertension was defined as systolic blood pressure ≥140 mmHg and/or diastolic blood pressure ≥90 mmHg and/or use of antihypertensive agents. ^c ^Past/current history of diabetus mellitus was defined as self-reported and/or hemoglobin _A__1c_ ≥5.8%. ^d^Decline in instrumental activities of daily living defined as scoring ≤10 in the Tokyo Metropolitan Institute of Gerontology Index of Competence. ^e^Depressed defined as scoring ≥2 in the Geriatric Depression Scale, 5-item version. ^f^Cognitive impairment defined as scoring ≤2 in the clock drawing test.

Table 2 shows the incidence and RRs of AHOs according to sensory impairments and gender. In both genders, vision impairment was related to an elevated risk of AHOs in both the crude analysis and in the vision and hearing mutually adjusted model. We observed no statistical evidence that the association between vision impairment and AHOs varied according to gender (p = .904 in the likelihood ratio test). Therefore, we performed combined-gender analyses for vision impairment. After adjusting for gender, age, education, marital status, smoking, alcohol consumption, past/current history of major illness and diabetes, we found that vision impairment was associated with AHOs (RR for vision impairment = 1.60, 95% CI = 1.05-2.44). On the other hand, the men with impaired hearing were 3 times more likely to experience AHOs than those without (RR for hearing impairment = 3.10, 95% CI = 1.43-6.72); no such increased risk was found among the women with impaired hearing. We found statistical evidence of interaction in the association of gender and hearing impairment with AHOs (p = .006 in the likelihood ratio test). Excluding events occurring within the first 1 year did not change the results substantially (data not shown).

**Table 2:** Risk ratios of adverse health outcomes (dependence in ADLs or death) for vision and hearing impairments by gender.

	Incidence (%)	Crude RR (95% CI)	*P*-value	Model 1 ^a ^ RR (95% CI)	*P*-value	Model 2 ^b ^ RR (95% CI)		*P*-value	*P *for interaction ^c^
Men									
Vision impairment									
No	23/275 (8.4)	1.00		1.00		1.00			
Impaired	11/60 (18.3)	2.19 (1.13-4.25)	.020	1.83 (0.95-3.53)	.071	1.43 (0.63-3.26)		.393	
Hearing impairment									
No	18/271 (6.6)	1.00		1.00		1.00			
Impaired	16/64 (25.0)	3.76 (2.03 -6.97)	< .001	3.49 (1.86-6.53)	< .001	3.10 (1.43-6.72)		.004	
Women									
Vision impairment									
No	23/301 (7.6)	1.00		1.00		1.00			
Impaired	29/160 (18.1)	2.37 (1.42-3.96)	.001	2.28 (1.35-3.83)	.002	1.60 (0.97-2.63)		.064	
Hearing impairment									
No	34/345 (9.9)	1.00		1.00		1.00			
Impaired	18/116 (15.5)	1.57 (0.93-2.68)	.094	1.40 (0.82-2.39)	.216	0.71 (0.38-1.31)		.269	
Men and Women									
Vision impairment									
No	46/576 (8.0)	1.00		1.00		1.00			
Impaired	40/220 (18.2)	2.28 (1.53-3.38)	< .001	2.07 (1.38-3.09)	< .001	1.60 (1.05-2.44)		.028	.904
Hearing impairment									
No	52/616 (8.4)	1.00		1.00		1.00			
Impaired	34/180 (18.9)	2.24 (1.50-3.34)	< .001	2.00 (1.33-3.01)	.001	1.23 (0.76-1.97)		.396	.006

RR: risk ratio, CI: confidence interval. a Variables included in the model 1 were vision and hearing impairments. ^b^In addition to the model 1, age (continuous), education, marital status, smoking, alcohol consumption, past/current history of major illness and diabetes mellitus were included. For combined-gender analysis, we further adjusted for gender.^ c^Statistical interaction between sensory impairments and gender with a likelihood ratio test.

As shown in Table 3, the RR for visual impairment in men was reduced by adjustment of three potential intermediate factors, while the RR in women remained little changed. With respect to hearing impairment, the RR in men was attenuated by 23% after adjusting for IADLs (RR = 2.39, 95% CI = 1.04-5.52). Adjustment for depressive mode attenuated the RR somewhat (RR = 2.72, 95% CI = 1.27-5.83), and for cognitive function only slightly (RR = 3.02, 95% CI = 1.37-6.62).

**Table 3:** Associations of vision and hearing impairments with adverse health outcomes (dependence in ADLs or death) through three potential intermediate factors by gender.

	Model 2 ^a ^ RR (95% CI)	Model 2 + Instrumental activities of daily living ^b ^ RR (95% CI)	*P*-value	Model 2 + depressive mode ^c ^ RR (95%) CI)	*P*-value	Model 2 + cognitive function ^d ^ RR (95% CI)	*P*-value
Men							
Vision impairment							
No	1.00	1.00		1.00		1.00	
Impaired	1.43 (0.63 3.26)	0.73 (0.24 2.21)	.578	1.20 (0.53 2.71)	.657	1.12 (0.50 2.52)	.784
Hearing impairment							
No	1.00	1.00		1.00		1.00	
Impaired	3.10 (1.43 6.72)	2.39 (1.04 5.52)	.041	2.72 (1.27 5.83)	.010	3.02 (1.37 6.62)	.006
Women							
Vision impairment							
No	1.00	1.00		1.00		1.00	
Impaired	1.60 (0.97 2.63)	1.56 (0.91 2.65)	.103	1.55 (0.89 2.68)	.119	1.60 (0.97 2.63)	.065
Hearing impairment							
No	1.00	1.00		1.00		1.00	
Impaired	0.71 (0.38 1.31)	0.75 (0.40 1.39)	.359	0.71 (0.37 1.34)	.290	0.70 (0.38 1.29)	.249

RR: risk ratio, CI: confidence interval. ^a^Variables included in the model 2 were vision and hearing impairments, age (continuous), education, marital status, smoking, alcohol consumption, past/current history of major illness and diabetes mellitus.^ b^Tokyo Metropolitan Institute of Gerontology Index of Competence (score 0-13). ^c^Geriatric Depression Scale, 5-item version (score 0-5). ^d^Clock drawing test (score 0-4).

In this population, 22 men (6.5%) and 14 women (3.0%) used hearing aids. Our findings were not altered substantially when we repeated all analyses after excluding these hearing aids users (data not shown).

## Discussion

In this 3-year cohort study of an older Japanese population, vision and hearing impairments were associated with AHOs, and the association appeared to vary by gender. In both genders, vision impairment was related to an elevated risk of AHOs with no significant differences between men and women. In contrast, a clear association between hearing impairment and AHOs was found only in the men.

The gender-specific differences observed in this study are not particularly surprising, since earlier studies have suggested gender differences in the association between sensory impairments and AHOs. In a study of the relationship between hearing impairment (assessed by the whispered voice test) and reduced survival, an association was observed in men but not in women [17]. As for the relationship between hearing impairment with AHOs, the National Health Interview Survey in the United States showed similar results to ours: self- or proxy-reported hearing impairment was an independent predictor of mortality for African American men, but not for women [33]. Another study that targeted only women showed that vision impairment (best corrected vision worse than 20/40) was related to functional decline, while hearing impairment (unable to hear 40 dB HL at 2 kHz in the better ear) was not [9]. These early studies support the results of our study, but it should be noted that assessment of gender differences was not their main objective.

The gender differences found in our study can be partially explained by the gender-specific social roles and characteristics typical of older Japanese. Retired Japanese men tend to withdraw from social activities [34], and hearing impairment may exacerbate their feelings of loneliness and estrangement from society. In contrast, most Japanese women of this generation are housewives whose everyday lives have been embedded in the local community from an early age [35]: their involvement in the local community may minimize the influence of communication problems caused by hearing impairment. On the other hand, vision impairment would greatly hinder the performance of housework, which would have a greater negative impact on the well-being of women, who usually do most of the housework. Our findings cannot, therefore, be generalized to other populations without further epidemiological research.

The potential mechanism by which gender-specific sensory impairments lead to AHOs can be analyzed according to the 3 earlier reported potential pathways [32]: 1) underlying conditions and risk factors such as smoking, alcohol consumption, obesity, hypertension and diabetes mellitus [36-40], which affect both sensory function and outcome; 2) a direct effect of sensory impairments on outcome; 3) an indirect effect of sensory impairments on outcome through some intermediate factors. As for the first pathway, we observed an attenuation of RR for sensory impairments after adjusting for underlying conditions and risk factors, indicating that this pathway is a valid factor in our results. To differentiate the second and the third pathways, IADLs, depression and cognitive function, which are all associated with sensory impairments [6,9,20,21,32], were adjusted for as potential intermediate factors. In men, the RR for hearing impairment was reduced by 23% and 12% after adjusting for IADLs and depressive mode, respectively. Thus, the relationship between hearing impairment and AHOs can be partially explained by this indirect pathway. After adjusting for the 3 potential intermediate factors, the RR for vision impairment in men was attenuated, but in women it remained unchanged. Vision impairment has been reported to be associated with increased risk of falls, hip fracture [41] and unintentional injury mortality [42]. Osteoporosis, a risk factor for hip fracture, is more prevalent in women than in men [13]. Thus, the pathway explaining the relationship between vision impairment and AHOs may vary between the genders. This issue needs to be addressed in future research involving large sample populations.

Our findings suggest that effective care of older adults with sensory impairments is important. Considering the high prevalence of hearing impairment in older populations [36,37], strategies to fight it are an important public health issue. Since recent evidence suggests that lifestyle factors such as smoking and chronic conditions such as hypertension and diabetes mellitus play a key role in the progression of age-related hearing loss [36-38,40,43], measures to address these issues should be begun in early adulthood. Proper use of technology can also minimize the impact of hearing impairment on quality of life [4]. The number of people with hearing impairment who use hearing aids is relatively small [14], so the simple promotion of hearing aid use could be a meaningful strategy in improving the well-being of older adults. With respect to vision, methods of preventing or delaying vision impairment already exist for the 5 major causes (uncorrected refractive error, cataract, glaucoma, diabetic retinopathy and macular degeneration) [39,44]. However, figures from the United Kingdom indicate that only 58.4% of adults aged 50 or older with vision problems actually receive quality care [45]. Consequently, efforts to educate people on the importance of seeking care for sensory impairments are indispensable. Our results also suggest that care related to sensory impairments should be targeted differently for men and women.

We were able to follow 99.4% of the subjects, so there is little possibility of selection bias caused by loss to follow-up. We also included a wide range of adverse health-related events as outcome items, and considered various covariates either as confounding factors or intermediate factors in AHOs. Moreover, studies using objective sensory measurements instead of subjects' self-reporting are very limited [8,9]. The limitation of this study, however, was that single examination on exposure might have underestimated the true association between sensory impairments and AHOs due to regression dilution effects. In addition, because of the small sample size, it was difficult to examine the relationships between sensory impairments and individual outcome (dependence in ADLs and death) and to exclude the possibility that the gender-specific associations between sensory impairments and AHOs were due to chance alone.

## Conclusion

This present study not only indicated a clear association between sensory impairments as measured by objective methods and AHOs in community-dwelling Japanese older adults, but also suggested the possibility of gender differences in the association. Gender-specific associations between sensory impairments and AHOs warrant further investigation.

## Competing interests

The authors declare that they have no competing interests.

## Authors' contributions

TM, YN and TT participated in the study design, data collection, analysis and interpretation of data, and writing of the article. YK, MN, SI, KA and AM participated in data collection and interpretation of data, and reviewed drafts of the article. KM, HS and SI participated in the study design as an otorhinolaryngologist or ophthalmologist, and reviewed drafts of the article. TO supervised data analysis and interpretation, and contributed to preparation of the article. All authors read and approved the final version of the article.

## Pre-publication history

The pre-publication history for this paper can be accessed here:

http://www.biomedcentral.com/1471-2318/9/50/prepub
